# Glycyrrhizin and its derivatives promote hepatic differentiation via sweet receptor, Wnt, and Notch signaling

**DOI:** 10.1016/j.bbrep.2021.101181

**Published:** 2021-12-04

**Authors:** Akihiro Morita, Yuta Omoya, Rie Ito, Yuya Ishibashi, Keiichi Hiramoto, Shiho Ohnishi, Nobuji Yoshikawa, Shosuke Kawanishi

**Affiliations:** a Department of Pharmaceutical Sciences, Suzuka University of Medical Science, Suzuka, Mie, 513-8670, Japan; b Matsusaka R&D Center, Cokey Co., Ltd., Matsusaka, Mie, 515-0041, Japan

**Keywords:** Liver regeneration, Hepatic differentiation, Sweet receptor, T1R3, Glycyrrhizin, HMGB1, high-mobility group box1, GL, glycyrrhizin, αGA, 18α-glycyrrhetinic acid, βGA, 18β-glycyrrhetinic acid, LSG, licorice saponin G, LSH, licorice saponin H, CBX, carbenoxolone, *3-O*-hemisuccinyl βGA, Mono, 3-*O*-mono-glucuronyl βGA, Glc, 3-*O*-[glucosyl (1 → 2)-glucuronyl] βGA, Api, 3-*O*-[apiosyl (1 → 2)-glucuronyl] βGA, DMSO, dimethyl sulfoxide, DP, (±)-2-(2,4-dichlorophenoxy) propionic acid, HNF-4α, hepatocyte nuclear factor 4α, AFP, α-fetoprotein, CK-19, cytokeratin 19, Hes, hairy and enhancer of split

## Abstract

The acute liver disease is involved in aberrant release of high-mobility group box 1 (HMGB1). Glycyrrhizin (GL), a traditional Chinese medicine for liver disease, binds to HMGB1, thereby inhibits tissue injury. However the mode of action of GL for chronic liver disease remains unclear.

We investigated the effects of glycyrrhizin (GL) and its derivatives on liver differentiation using human iPS cells by using a flow cytometric analysis.

GL promoted hepatic differentiation at the hepatoblast formation stage. The GL derivatives, 3-*O*-mono-glucuronyl 18β-glycyrrhetinic acid (Mono) and 3-*O*-[glucosyl (1 → 2)-glucuronyl] 18β-glycyrrhetinic acid increased AFP^+^ cell counts and albumin^+^ cell counts. Glucuronate conjugation seemed to be a requirement for hepatic differentiation. Mono exhibited the most significant hepatic differentiation effect.

We evaluated the effects of (±)-2-(2,4-dichlorophenoxy) propionic acid (DP), a T1R3 antagonist, and sucralose, a T1R3 agonist, on hepatic differentiation, and found that DP suppressed Mono-induced hepatic differentiation, while sucralose promoted hepatic differentiation. Thus, GL promoted hepatic differentiation via T1R3 signaling. In addition, Mono increased β-catenin^+^ cell count and decreased Hes5^+^ cell count suggesting the involvement of Wnt and Notch signaling in GL-induced hepatic differentiation.

In conclusion, GL exerted a hepatic differentiation effect via sweet receptor (T1R3), canonical Wnt, and Notch signaling.

## Highlights

• Glycyrrhizin (GL) derivatives increased AFP^+^ and albumin^+^ cell counts.
• Glycyrrhizin promotes hepatic differentiation at the hepatoblast formation stage.
• Mono glucuronide showed the most significant hepatic differentiation effect.
• Glycyrrhizin promotes hepatic differentiation via T1R3 signaling.
• Wnt and Notch signaling are involved in GL-induced hepatic differentiation.

## 1 Introduction

Liver disease and its complications are a global health concern. There are many types of liver diseases, e.g., viral hepatitis, alcoholic and nonalcoholic liver diseases, cholangiopathies, autoimmune liver disease, which may lead to advanced liver disease stages, cirrhosis or hepatic malignancies. Liver disease and cirrhosis are significant health disorders with limited therapeutic strategies available for their management. Aberrant extracellular release of high-mobility group box1 (HMGB1) is associated with many forms of liver disease [1].

Licorice (*Glycyrrhiza* species) is an important medicinal herb. In traditional Chinese medicines, it has been shown to exhibit several pharmacological effects against inflammation, oxidative stress, immune disorders, virus infections, and cancer [[2], [3], [4], [5]]. More specifically, licorice is commonly used in the treatment of liver diseases, such as acute and chronic liver disease, hepatic steatosis, liver fibrosis, and hepatomas [2]. Approximately 400 major bioactive compounds have been identified in *Glycyrrhiza* species. Among them, triterpene saponins, glycyrrhizin (GL) and its derivatives are the most abundant. After oral administration of GL, it is irreversibly transformed by gut microbiota into glycyrrhetinic acid (GA), which is an aglycon of GL [2]. Thus, GL is thought to elicit the same pharmacological effects *in vivo* as GA. Actually, GL and GA bind to HMGB1 [4] and significantly inhibit tissue injury [5]. In our preliminary study, as opposed to GA, GL was found to promote hepatic differentiation. The liver is constituted of 2 major epithelial cell types, which are hepatocytes and cholangiocytes and are derived from hepatoblasts, fetal liver stem/progenitor cells [6,7]. Thus, the effects of GL on cellular differentiation was investigated by flow cytometric measurement of the expression levels of several marker proteins, including cytokeratin-19 (CK19), a cholangiocyte marker, albumin, a mature hepatocyte marker; and α-fetoprotein (AFP), a hepatic stem cell marker [8].

High doses of several compounds have been used in *in vitro* studies, e.g., in a previous study, the effects of Fuzhenghuayu, at a concentration of 50–100 μg/mL, on hepatic differentiation were evaluated [9]. In another study, the anti-hepatitis C effects of GL at dose of 30–1000 μM [10], and its anti-SARS-CoV effects at an EC_50_ of 0.44 mg/mL (534 μM) were evaluated [11]. In addition, after the administration of a single dose of shakuyakukanzoto (2.5 g), containing 26 mg of GL (31.59 μmol), in healthy human volunteers, its maximal plasma concentration was found to be 100 ng/mL (213 nM) [12]. Human iPS cells have been extensively used in drug discovery and for clarifying the pharmacological mechanisms of drug action [13,14]. Considering the clinical effectiveness of GL, we investigated its effects at nM-scale concentration on cell differentiation using human iPS cell technology.

In this study, we found that glycyrrhizin promotes hepatic differentiation and involvement of a sweet receptor and Wnt-Notch signaling.

## 2 Materials and methods

### 2.1 Materials

α- and β-glycyrrhetinic acid (GA), and carbenoxolone, 3-*O*-hemisuccinyl βGA were purchased from Merck, Germany, glycyrrhizin (GL) and 3-*O*-mono-glucuronyl βGA were purchased from Nagara Science Co., Japan, licorice saponin G was purchased from Cokey Co., Japan, (±)-2-(2,4-dichlorophenoxy) propionic acid was purchased from FUJIFILM Wako Pure Chemical, Japan, and sucralose was purchased from Combi-Blocks, USA. 3-*O*-[glucosyl (1 → 2)-glucuronyl] βGA (Glc) was isolated from the roots of *Glycyrrhiza uralensis* strain 83-555, according to a method previously described by Cokey Co [15]. 3-*O*-[apiosyl (1 → 2)-glucuronyl] βGA (Api) and licorice saponin H were isolated from commercially available licorice extracts using a method similar to that used for the extraction of Glc [16]. These compounds were identified by comparing their spectral data with published data.

### 2.2 Cell culture and hepatic differentiation

For the maintenance culture, human induced pluripotent stem cells (iPS, 201B7 line, Riken, Japan) were subcultured on a mitomycin-treated-feeder layer (SNL 76/7 cells, ECACC, UK) in an hES medium (DMEM/F12 [FUJIFILM Wako Pure Chemical], 20% Knockout serum replacement [Thermo Fisher Scientific, USA], 2 mM glutamine [Thermo Fisher Scientific], 0.1 mM non-essential amino acids [Thermo Fisher Scientific], 50 U penicillin and 50 mg/mL streptomycin [Thermo Fisher Scientific], and 100 nM 2-mercaptoethanol [Merck] supplemented with 4 ng/mL basic fibroblast growth factor (bFGF) [FUJIFILM Wako Pure Chemical]) in a 5% CO_2_ humidified atmosphere according to the method described by Takahashi et al. [17]. The medium was changed daily.

**Fig. 1:**
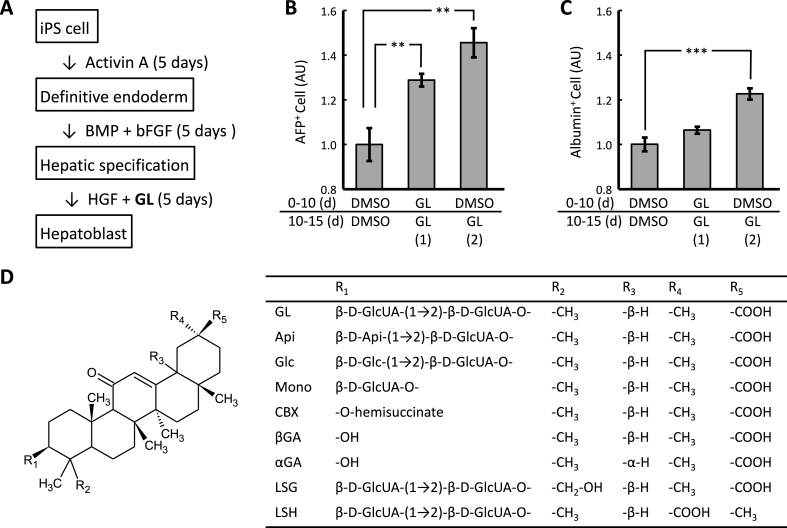
Effects of glycyrrhizin on hepatic differentiation of human iPS cells. Hepatic induction of human iPS cells was performed according to the method described by Si-Tayeb et al. [18] with minor modification (A). After immunoreaction with anti-APF (B) and anti-albumin (C) antibodies, the cells were subjected to flow cytometric analysis. ** and *** indicate significant differences (*p* < 0.01 and 0.001, respectively) as determined by ANOVA followed by Tukey's post-hoc test (*n* = 3). DMSO: solvent control; GL: 1 μM glycyrrhizin treatment; (1): GL treatment throughout the hepatic differentiation; (2): GL treatment for the hepatoblast formation stage. Structure of glycyrrhizin and its derivatives (D). GL: glycyrrhizin; αGA: 18α-glycyrrhetinic acid; βGA: 18β-glycyrrhetinic acid; LSG: licorice saponin G; LSH: licorice saponin H; CBX: carbenoxolone, 3-*O*-hemisuccinyl βGA; Mono: 3-*O*-mono-glucuronyl βGA; Glc; 3-*O*-[glucosyl (1 → 2)-glucuronyl] βGA; Api: 3-*O*-[apiosyl (1 → 2)-glucuronyl] βGA.

We induced hepatic differentiation, according to the method described by Si-Tayeb et al. [18] with minor modification (Fig. 1A). In brief, iPS cells dissociated using accutase (Innovative Cell Technologies, USA) were seeded at a concentration of 1 × 10^4^ cells/cm^2^ onto a Matrigel (GFR) (Corning, USA)-coated dish and cultured in an hES medium which was changed daily. To induce the differentiation of the definitive endoderm, 7 days after culturing (on day 0), the medium was replaced with an RPMI medium (RPMI 1670 [Nakarai tesque, Japan] containing 2% B27 supplement [Thermo Fisher Scientific], 2 mg/mL l-glutamine [Thermo Fisher Scientific], 50 U penicillin and 50 mg/mL streptomycin [Thermo Fisher Scientific]) supplemented with 100 ng/mL activin A (Shenandoah Biotechnology, USA) and changed every other day. To induce hepatic specification, the medium was replaced with an RPMI medium supplemented with 10 ng/mL bFGF and 20 ng/mL bone morphogenetic protein 4 (BMP4, Humanzyme, USA) on day 5 and changed every other day. Then, for hepatoblast formation, the medium was replaced with an RPMI medium supplemented with 20 ng/mL hepatocyte growth factor (HGF, Humanzyme) on day 10 and changed every other day. GL and its derivatives were added on days 10–15, except otherwise mentioned.

### 2.3 Flow cytometric analysis

Cultured cells were dissociated using dispase (Thermo Fisher Scientific) and a cell dissociation buffer (Thermo Fisher Scientific). Cells fixed with Cytofix Fixation Buffer (BD, USA) were washed with PhosFlow Perm/Wash Buffer I (BD) and incubated with primary antibodies (anti-HNF4α [GeneTex, USA], anti-CK19 [GeneTex], anti-AFP [R&D Systems, USA], anti-albumin [R&D Systems], anti-β-catenin [GeneTex], anti-Numb [R&D Systems], anti-Notch 1 ICD [R&D Systems], and anti-Hes5 [Bioss, USA] antibodies at a dilution of 1:400) at 4 °C overnight. After the cells were washed, they were incubated with Alexa 488 (Cell Signaling Technology, USA)- and phycoerythrin (Southern Biotech, USA)-conjugated anti-rabbit and anti-mouse IgG, respectively at 4 °C overnight. Then, the cells were washed again and passed through a 45-μm filter, and were subjected to flow cytometric analysis using a FACS Calibur (BD) flow cytometer according to the manufacturer's instructions. For flow cytometric analysis, the proportion of immunopositive cells was determined using the antibodies mentioned above. The data, which were normalized to the population of HNF4α^+^ cells, were represented as the relative value compared to that obtained following solvent control treatment.

### 2.4 Statistical analysis

Several statistical tests such as ANOVA followed by Tukey's post-hoc test, Student's *t*-test with a significance level of 0.05, and Fisher's protected least significant difference (LSD) test with a significance level of 0.025, were performed.

## 3 Results

### 3.1 Glycyrrhizin promoted hepatic differentiation

To determine whether the liver regeneration effects of GL are due to the promotion of liver differentiation, in preliminary experiment, we induced hepatoblasts in a medium containing 1 μM GL throughout the culture period. Treatment with GL increased AFP^+^ cell count and slightly increased albumin^+^ cell count (data not shown). In addition, to determine the sensitive period of the cells to GL, the duration of treatment was modified as follows: (1) throughout the hepatic differentiation, for 15 days, or (2) only for the hepatoblast formation stage, i.e., the last 5 days of hepatic differentiation. AFP^+^ cell count significantly increased by GL treatment (*p* < 0.01, ANOVA followed by Tukey's post-hoc test, *n* = 3, Fig. 1B). Moreover the stage-restricted treatment was significantly more effective than GL treatment throughout differentiation. The GL treatment for the last 5 days significantly increased albumin^+^ cell count (*p* < 0.001, ANOVA followed by Tukey's post-hoc test, *n* = 3, Fig. 1C). Therefore we concluded that GL promoted hepatic differentiation.

### 3.2 Structure-activity relationship between glycyrrhizin and its derivatives and their hepatic differentiation effects

**Fig. 2:**
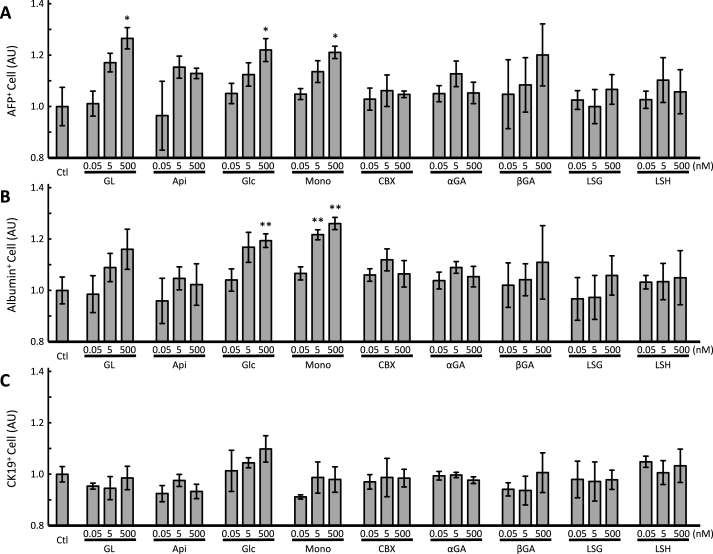
Effect of glycyrrhizin and its derivatives on hepatic differentiation of human iPS cells. Glycyrrhizin (GL), its derivatives (Api, Glc, Mono, CBX, αGA, βGA, LSG, and LSH), or the solvent control (Ctl) were administered to cells at the hepatoblast formation stage (on days 10–15). After immunoreaction with anti-APF (A), anti-albumin (B), and anti-CK-19 (C) antibodies, the cells were subjected to flow cytometric analysis. * and ** represent significant differences compared to Ctl (*p* < 0.025 and 0.005, respectively) as determined by Fisher's protected LSD (*n* = 3).

To clarify the mode of action of GL, we investigated the structure (Fig. 1D) and activity (Fig. 2, Supplemental Fig. A-C) relationships between it and its 8 derivatives. As concerns aglycones, βGA showed hepatic differentiation activity in a dose-dependent manner. However, there were no significant differences between βGA at a concentration of 500 nM and control (*p* = 0.11496, Fisher's protected LSD, *n* = 3). Another aglycon, αGA, had no effect on hepatic differentiation. CBX, 3-*O*-hemisuccinyl βGA, also had no effect on hepatic differentiation. The glucuronate conjugates, GL, Mono, and Glc showed hepatic differentiation activity in a dose-dependent manner, and significantly increased AFP^+^ cell count at a concentration of 500 nM (*p* = 0.00628, 0.01387 and 0.01624, respectively, Fisher's protected LSD, *n* = 3). Api tended to increase AFP^+^ cell count at a concentration of 5 and 500 nM. However, LSG and LSH had no effects on hepatic differentiation. No aglycone nor CBX had an effect on albumin^+^ cell count. In contrast, GL increased albumin^+^ cell count in a dose-dependent manner. Moreover, Glc and Mono significantly increased albumin^+^ cell count at a concentration of 500 nM (Glc and Mono, *p* = 0.00458 and 0.00069, respectively; Fisher's protected LSD, *n* = 3) and 5 nM (Mono, *p* = 0.00182, Fisher's protected LSD, *n* = 3). Furthermore, GL and all its derivatives, had no effect on CK19^+^ cell count, indicating that their effects were restricted to hepatocyte differentiation, as they did not influence cholangiocyte differentiation. Moreover, these effects were observed at low concentrations, similar to plasma concentrations obtained after a single oral administration [12]. Mono exhibited the most significant hepatic differentiation effects; however, it did not affect cholangiocyte differentiation. These results indicated that the structure of Mono allows for interaction with specific receptors for the induction of hepatic differentiation.

### 3.3 The sweet receptor is involved in glycyrrhizin-induced hepatic differentiation

**Fig. 3:**
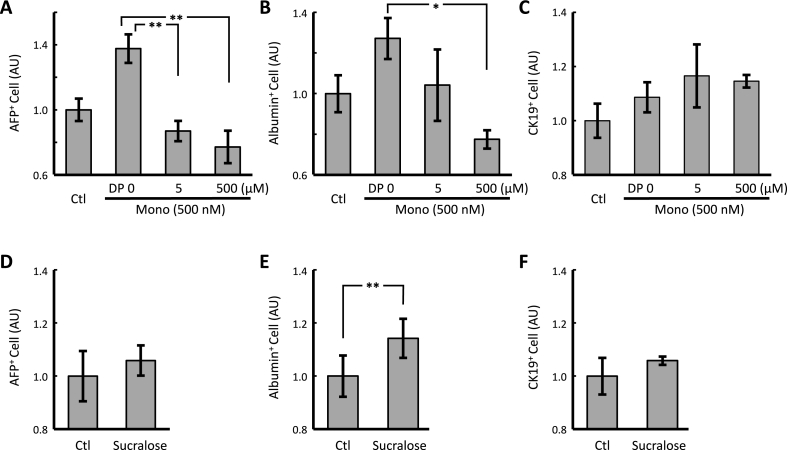
Effect of a sweet receptor agonist and antagonist on glycyrrhetinic acid mono glucuronide-induced hepatic differentiation of human iPS cells. Hepatic induction of human iPS cells was performed. Mono and DP (T1R3 antagonist; A-C), sucralose (T1R3 agonist; D-F), or the solvent control (Ctl) was administered to the cells at the hepatoblast formation stage (on days 10–15). After immunoreaction with anti-APF (A, D), anti-albumin (B, E), and anti-CK-19 (C, F) antibodies, the cells were subjected to flow cytometric analysis. * and ** indicate significant differences (*p* < 0.025 and 0.005, respectively) as determined by Fisher's protected LSD (*n* = 3).

Mizutani et al. [19] investigated the taste profiles of GL and a variety of 3-*O*-glycosides, and found that Mono exhibited the highest sweetness level. The relationship between structure and sweetness found in their study is similar to the relationship between structure and haptic differentiation activity found in this study. Actually, GL has been found to stimulate the sweet receptor, T1R3, in pancreatic β-cells [20]. Therefore, we investigated the effects of DP and sucralose, which are an effective inhibitors [21] and agonists [20], respectively, of T1R3, on hepatic differentiation (Fig. 3). Treatment with DP significantly decreased Mono-induced AFP^+^ cell count (5 and 500 μM, *p* = 0.00464 and 0.00522, respectively, Fisher's protected LSD, *n* = 3) and significantly decreased albumin^+^ cell count at a concentration of 500 μM in a dose-dependent manner (*p* = 0.01027, Fisher's protected LSD, *n* = 3). Treatment with 50 μM sucralose significantly increased albumin^+^ cell count (*p* = 0.00771, *t*-test, *n* = 3). Thus, these results strongly suggest that the hepatocyte differentiation effects of GL involve in T1R3.

### 3.4 Beta-catenin-Notch signaling is involved in glycyrrhizin-induced hepatic differentiation

**Fig. 4:**
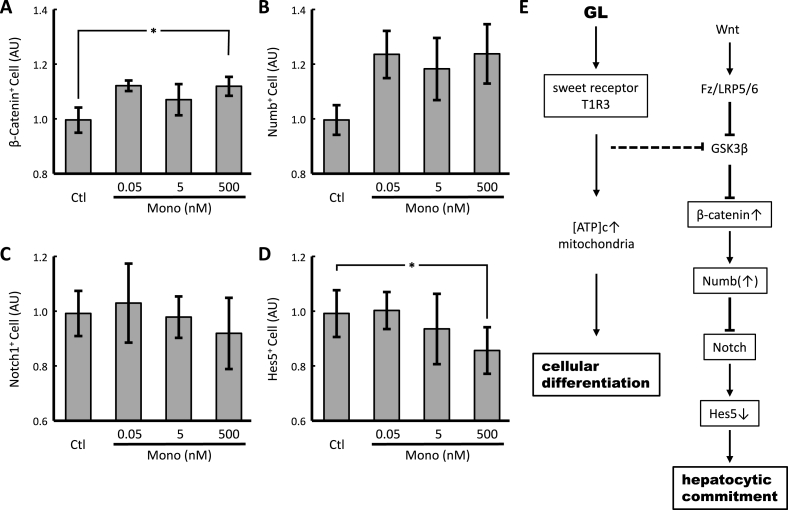
Effect of glycyrrhetinic acid mono glucuronide on Wnt and Notch signaling in hepatic differentiation of human iPS cells. Mono or the solvent control (Ctl) was administered to the cells at the hepatoblast formation stage (on days 10–15). After immunoreaction with anti-β-catenin (A), anti-Numb (B), anti-Notch1 (C) and anti-Hes5 (D) antibodies, the cells were subjected to flow cytometric analysis. * indicates significant differences (*p* < 0.025) as determined by Fisher's protected LSD (*n* = 3–4). The GL signaling pathway during liver regeneration (E). [ATP]c: intracellular ATP concentration; Fz/LRP5/6: Frizzled/low-density lipoprotein receptor-related protein5/6; GSK3β: glycogen synthase kinase 3β.

As mentioned above, GL promoted hepatic differentiation but not cholangiocyte differentiation. The fate of hepatoblasts whether they be hepatocytes or cholangiocytes is determined by Notch signaling. Notch signaling and its downstream transcription factors, Hes1 and Hes5, are crucial for cholangiocyte differentiation [8,9,22]. Recently, Boulter et al. [22] reported that canonical Wnt signaling maintains the expression of Numb, which is a Notch inhibitor, in hepatoblasts; thus, Wnt promotes specification to hepatocytes. Therefore, we evaluated whether Wnt, or Notch signaling or both were involved in GL-induced hepatic differentiation (Fig. 4, Supplemental Fig. D-G). β-catenin and Numb are important factors in the canonical Wnt signaling pathway. Treatment with 500 nM Mono significantly increased β-catenin^+^ cell count (*p* = 0.01196, Fisher's protected LSD, *n* = 3). Although there is no significant difference in Numb^+^ cell count between Mono treatment and control (at 500 nM, *p* = 0.03076, Fisher's protected LSD, *n* = 3), it increased by Mono treatment. In contrast, Notch^+^ cell count was not affected; however, the same treatment significantly decreased Hes5^+^ cell count (*p* = 0.00905, Fisher's protected LSD, *n* = 4). Thus, Mono, a GL derivative, was found to stimulate canonical Wnt signaling and to suppress Notch signaling during hepatic differentiation.

## 4 Discussion

In this study, we clarified that GL and some of its derivatives promote hepatic differentiation. At present, GL has been shown to have various effects on liver disease, including anti-oxidative stress, anti-inflammatory and immunoregulatory, anti-viral, anti-steatosis, anti-liver fibrosis and anti-cirrhosis, and anti-cancer effects [,^23^]. Although Kimura et al. [24] reported that GL enhanced the regeneration of liver mass and function in 70% partially hepatectomized rats, its effects on hepatic regeneration have not been clearly elucidated. Hepatic regeneration has been proposed to occur in 2 scenarios, i.e., during acute and chronic hepatic injury [8,9,25]. During acute cellular injury, hepatocyte proliferation is increased to reconstitute the damaged tissue. During chronic hepatocellular injury, almost all hepatocytes are senescent, and non-senescent periportal hepatocyte and expanded biliary epithelial cells contribute to hepatocyte regeneration. These hepatocytes can differentiate into biliary cells, and back into hepatocytes. The plasticity of hepatocytes and biliary cells enhances liver regeneration via an intermediate transition through biopotential biliary epithelial cells, which have been proposed as liver progenitor cells. Therefore, liver regeneration in chronic injury may recapitulate liver development [8,9]. In this study, we found that GL may be beneficial in the treatment of chronic liver disease as it promotes hepatic differentiation.

As shown in Fig. 1-D, we found that GL, Mono and Glc were effective in inducing hepatic differentiation. These results indicate that the hepatic differentiation effects of these saponins originated from structures consist of a βGA, an aglycone, conjugated with 1 or 2 glucuronate at C3. Lack of hepatic differentiation effect with LSH and LSG suggests following assumptions for the structure-activity relationship: (1) The position of COOH group, i.e., C30 at the aglycone region is critical; (2) Hydroxylation of C24 at the region abolishes the effect of GL. In addition, the hepatic differentiation effect depends on the types of second sugar moieties, glucuronate, glucose and apiose, respectively: though glucose, a neutral sugar with pyranose ring, enhanced the activity, apiose, a neutral sugar with furanose ring, inactivated it. These results suggest that a sugar consist of pyranose is preferable for the second sugar.

The relationship between structure and sweetness [19] is similar to the relationship between structure and hepatic differentiation activity found in this study. Sweetness is perceived by means of the sweet receptor, T1R3 [26]. DP, a T1R3 antagonist inhibits the effects of Mono. Sucralose, a T1R3 agonist, has similar effects to those of GL, indicating that the effects of GL on hepatic differentiation involve T1R3. T1R3 exists not only in taste cells but also in the intestine, pancreas, liver, and adipose tissue [27]. In pancreatic β-cells, GL and sucralose induce an elevation of intracellular ATP levels even in the absence of ambient glucose, suggesting that sucralose acts on the mitochondrion and promotes metabolism [20,21]. Mitochondrial dynamics play an important role in cellular differentiation and maintenance of stemness. The predominant energy metabolism pathways in several stem cells and in differentiated cells are glycolysis and oxidative phosphorylation, respectively [28]. Actually, Nakagawa et al. [29] reported that maintaining a high ATP concentration in the liver tissues enhanced liver regeneration. Moreover, mitochondrial remodeling occurs in hepatic differentiation and dedifferentiation [30]. Thus, GL may promote cellular differentiation by modifying mitochondrial dynamics via T1R3.

There are 2 predominant cell types in the liver, hepatocytes and cholangiocytes. The fate of these 2 cell types depend on Wnt and Notch signaling [22]. During canonical Wnt signaling, β-catenin maintains the expression of Numb, a Notch inhibitor, in hepatoblasts, and Notch signaling promotes the biliary specification of hepatic progenitor cells to cholangiocytes [8,9,22]. In this study, Mono increased β-catenin^+^ cell count, and decreased Hes5^+^ (a Notch target) cell count. These results indicate that Mono activates canonical Wnt signaling and suppresses Notch signaling, which is required for cholangiocyte commitment. Ahmad et al. [31] reported that GL mediates the downregulation of the Notch pathway in cervical cancer cells. Furthermore, Bai et al. [32] reported that GL promotes osteogenic differentiation of bone marrow stromal cells by activating Wnt signaling. Tian et al. [33] showed that in oligodendrocyte progenitor cells, GL suppresses the expression of glycogen synthase kinase 3β, which degrades β-catenin in the canonical Wnt signaling pathway. Although GL may directly stimulate Wnt signaling, our data indicate that it exerts its effects on hepatocyte differentiation via T1R3. Collectively, GL promotes cellular differentiation by regulating mitochondrial dynamics via T1R3, and commits the fate of hepatocytes by inhibiting Notch signaling inhibition via the activation of canonical Wnt signaling (Fig. 4E). The clarification of the pathway between T1R3 and Wnt signal remains for the future study.

## Declaration of competing interest

The authors report no conflicts of interest.

## Appendix A Supplementary data

Multimedia component 1 Representative flow cytometry profiles and cell images. Mono or the solvent control (Ctl) was administered to the cells at the hepatoblast formation stage (on days 10–15). After immunoreaction with anti-AFP (A), anti-albumin (B), and anti-CK-19 (C), anti-β-catenin (D), anti-Numb (E), anti-Notch1 (F) and anti-Hes5 (G) antibodies, the cells were subjected to flow cytometric analysis. The remained sample were stained with Hoechist 33342 (1 μg/mL, 20 min) for nuclear staining. Cells were concentrate onto a microscopic slide by using the Cytofuge cytocentrifuge (IRIS International, Inc., USA) and observed by a fluorescent microscopy (Olympus, Japan). Multimedia component 1
