# Supplementary figures and images for: Glycyrrhizin and its derivatives promote hepatic differentiation via sweet receptor, Wnt, and Notch signaling
Source: Biochem Biophys Rep. 2021 Dec 4;28:101181. doi: 10.1016/j.bbrep.2021.101181 (PMC8654616; doi:10.1016/j.bbrep.2021.101181)

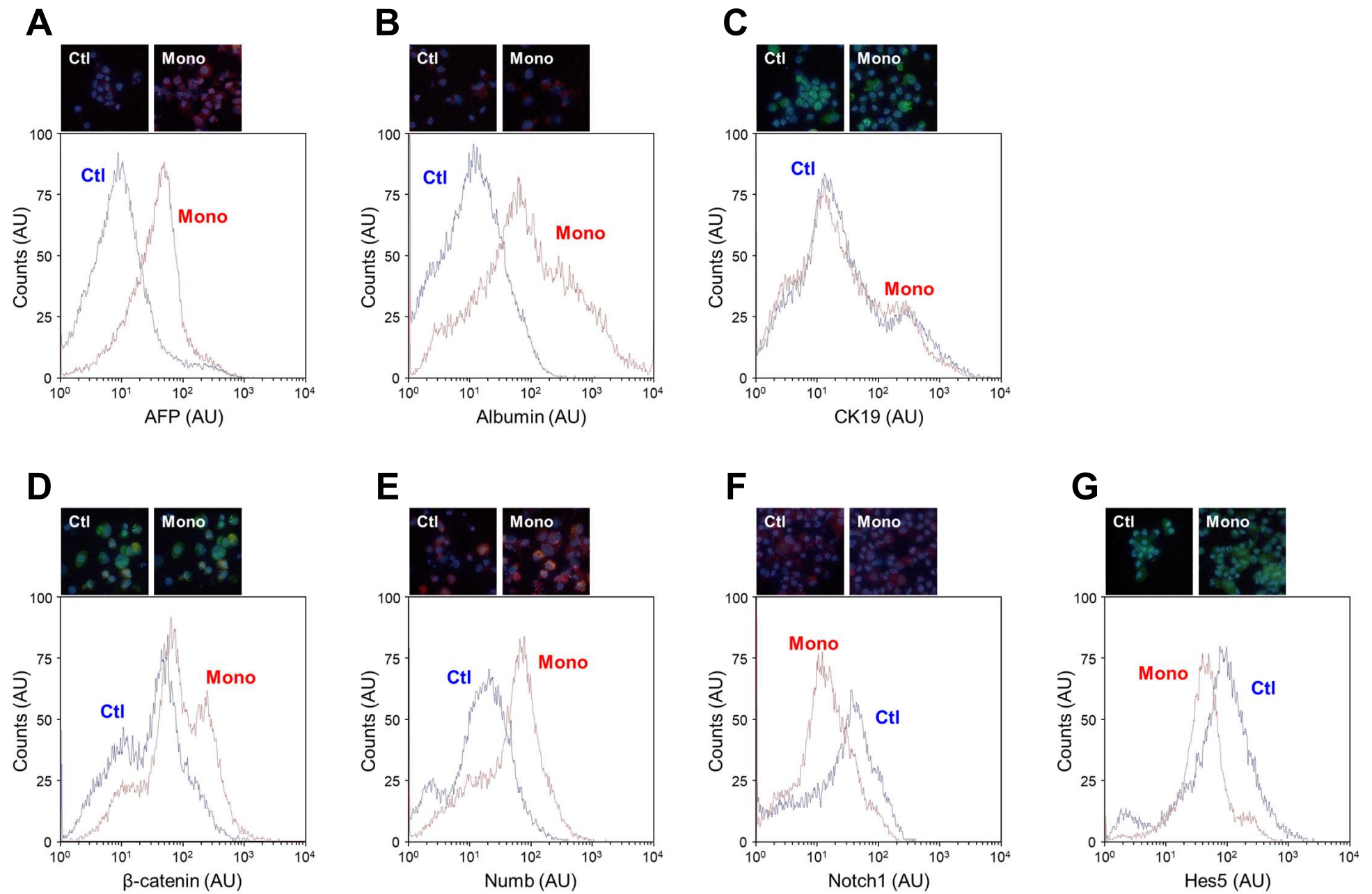

Supplement: Multimedia component 1 — Representative flow cytometry profiles and cell images. Mono or the solvent control (Ctl) was administered to the cells at the hepatoblast formation stage (on days 10–15). After immunoreaction with anti-AFP (A), anti-albumin (B), and anti-CK-19 (C), anti-β-catenin (D), anti-Numb (E), anti-Notch1 (F) and anti-Hes5 (G) antibodies, the cells were subjected to flow cytometric analysis. The remained sample were stained with Hoechist 33342 (1 μg/mL, 20 min) for nuclear staining. Cells were concentrate onto a microscopic slide by using the Cytofuge cytocentrifuge (IRIS International, Inc., USA) and observed by a fluorescent microscopy (Olympus, Japan). [file mmc1.pdf]
